# Reducing Postoperative Complications in High-Risk Breast Surgery Patients: A Preliminary Study on the Efficacy of NPWT Dressing

**DOI:** 10.3390/jpm15030104

**Published:** 2025-03-06

**Authors:** Raquel Diaz, Ilaria Baldelli, Letizia Cuniolo, Ludovico Ponzielli, Elisa Bertulla, Giada Marassi, Federica Murelli, Chiara Cornacchia, Francesca Depaoli, Cecilia Margarino, Chiara Boccardo, Marco Gipponi, Simonetta Franchelli, Marianna Pesce, Franco De Cian, Piero Fregatti

**Affiliations:** 1 Department of Surgical and Diagnostic Integrated Sciences (DISC), University of Genova, 16132 Genova, Italy4188856@studenti.unige.it (E.B.);; 2 Plastic and Reconstructive Surgery Unit, San Martino Polyclinic Hospital, 16132 Genova, Italy; 3 School of Medicine, University of Genoa, 16126 Genoa, Italy; 4 Breast Surgery Unit, IRCCS Ospedale Policlinico San Martino, 16132 Genova, Italy; chiara.cornacchia@hsanmartino.it (C.C.);

**Keywords:** breast cancer, oncoplasty, mastectomy, NPWT dressing

## Abstract

**Background**: Negative Pressure Wound Therapy (NPWT) has proven to be an effective intervention in preventing postoperative complications across a range of surgical specialties, including orthopedics, vascular, and abdominal surgery. This study aimed to assess the prophylactic use of NPWT dressing compared to the Standard of Care (SOC) in high-risk patients undergoing oncoplastic and reconstructive breast surgery. **Materials and Methods**: This preliminary case-control study included 23 high-risk patients, enrolled between September 2023 and February 2024, at San Martino Polyclinic Hospital, Genoa. High-risk patients were defined as those with one or more of the following risk factors: obesity, prior radiotherapy, neoadjuvant chemotherapy, smoking history, diabetes, or corticosteroid use. The surgical procedures evaluated in this study included mastectomy with immediate implant-based breast reconstruction, reduction mammoplasty, and oncoplastic breast surgery following local excision or quadrantectomy. NPWT dressing was applied immediately after skin closure in the operating room, replaced after 2–3 days, and removed 7 days post-procedure. Surgical outcomes assessed included skin flap necrosis, wound dehiscence, infection, implant loss, and delays in adjuvant therapy. **Results**: A total of 23 patients, aged 45 to 57 years, were enrolled. Eleven patients received NPWT dressing, while twelve were treated with SOC. No complications occurred in the NPWT dressing group, whereas four complications were observed in the SOC group. Of the control group, three patients developed infections, which were treated with oral antibiotics for two, while one required implant replacement surgery. The remaining patient in the control group experienced wound dehiscence, which was successfully managed conservatively on an outpatient basis. **Discussion and Conclusions**: Our findings suggest that prophylactic NPWT dressing in oncoplastic and reconstructive breast surgery results in a significantly lower rate of wound-related complications. Although this is a preliminary study, it provides a foundation for further research in a larger cohort. These results also prompt a discussion of the cost-effectiveness of NPWT dressing relative to the SOC, given its higher cost.

## 1. Introduction

Breast cancer remains one of the most prevalent malignancies affecting women worldwide, posing a substantial public health challenge. It is estimated that 1 in 8 women will develop breast cancer during their lifetime, underscoring the importance of early detection, personalized treatments, and innovative surgical techniques to improve survival rates. While advancements in treatment strategies, including targeted therapies, immunotherapies, and precision medicine, have significantly reduced mortality, breast cancer remains a leading cause of death among women, particularly in cases of late-stage diagnosis or recurrence [1,2,3].

Surgical intervention remains a cornerstone in breast cancer management, particularly for patients with localized tumors. Mastectomies, breast-conserving surgeries (e.g., lumpectomies), and oncoplastic reconstructions are essential components of treatment, aiming to remove malignant tissue while preserving both the aesthetic and functional integrity of the breast [4]. However, these procedures carry inherent risks, including postoperative complications such as infections, delayed healing, seromas, and necrosis, which can adversely affect recovery and compromise patients’ quality of life [5,6,7].

In the era of personalized medicine, post-surgical care plays a critical role in optimizing functional and aesthetic outcomes. Negative Pressure Wound Therapy (NPWT) has emerged as a transformative approach for managing surgical wounds, especially in complex surgeries such as breast cancer procedures. This therapy uses a controlled vacuum system to apply negative pressure to the wound site, enhancing tissue oxygenation, reducing fluid buildup, and stimulating tissue regeneration [8]. NPWT systems have demonstrated considerable promise in breast cancer surgeries by providing a non-invasive and convenient solution to mitigate complications and accelerate recovery [9].

The NPWT dressing is specifically designed to maintain a sealed environment around the wound while applying consistent negative pressure of −80 mmHg. This mechanism aids in removing excess exudate, reducing seroma formation, and minimizing infection risks by isolating the wound from external contaminants. Additionally, it enhances microcirculation and tissue perfusion, critical factors for optimal wound healing. By promoting cell proliferation, angiogenesis, and granulation tissue formation, NPWT accelerates healing, reduces scarring, and improves cosmetic outcomes for patients [10,11].

This approach is particularly beneficial for high-risk patients, including those with obesity, diabetes, smoking history, or those who have undergone chemotherapy or radiotherapy—factors that can impair natural wound healing and increase susceptibility to complications such as wound dehiscence and infections. For these patients, NPWT dressing offers an effective strategy to enhance healing times, minimize scarring, and improve surgical outcomes [12,13].

This study aims to evaluate the effectiveness of the NPWT dressing in managing postoperative wounds in breast cancer patients, particularly in preventing complications like infections, seromas, and delayed healing. By exploring the molecular and cellular responses to NPWT in a high-risk cohort, we aim to provide evidence for the system’s integration into personalized postoperative care for breast cancer surgeries.

## 2. Materials and Methods

The study was conducted from December 2023 to May 2024, spanning a total of 5 months and involving patients from the Breast Surgery Department of San Martino Hospital in Genoa.

Since the PICO system is already integrated into standard care protocols, its use does not constitute a clinical trial or experimental procedure. For this reason, it does not require prior approval from the ethical committee.

This prospective exploratory study aimed to evaluate the efficacy of negative pressure wound therapy (NPWT) dressings in reducing postoperative complications and enhancing wound healing outcomes in high-risk patients undergoing oncologic-reconstructive breast procedures.

A total of 22 patients were enrolled and divided equally into two groups: a control group receiving standard postoperative dressings, and an experimental group treated with NPWT dressings. The experimental group signed consent for the use of their data and the application of the PICO dressing. Patients were included based on their risk profiles, which aligned with established criteria from the Association of Breast Surgery (ABS) [14] and the British Association of Plastic, Reconstructive, and Aesthetic Surgeons (BAPRAS) [15]. The mean age of the patients was 51.5 years (range: 33–69 years), and all participants were female. Clinical characteristics, including body mass index (BMI), smoking status, comorbidities, and prior treatments, were systematically documented to provide a comprehensive profile of the cohort.

Inclusion criteria focused on high-risk patients scheduled for oncologic-reconstructive surgeries. High-risk factors included being overweight (BMI 25–29.9 kg/m^2^), obesity (BMI ≥ 30 kg/m^2^), smoking, diabetes mellitus (Type I or II), history of radiotherapy, neoadjuvant chemotherapy, corticosteroid therapy, or previous surgeries on the affected breast. Patient-specific anatomical factors, such as thin skin, were also evaluated, as thin skin is particularly prone to wound-related complications. To assess subcutaneous thickness, the skin pinch test was performed. This method involves gently pinching the skin at the surgical site to measure the thickness of the subcutaneous layer. The skin pinch test provides a simple and reliable assessment of skin fragility and helps identify patients at higher risk for complications such as wound dehiscence or ischemia.

Patients undergoing purely oncologic procedures, such as simple mastectomies or quadrantectomies without reconstructive elements, were excluded. This ensured the study focused on the combined challenges of oncologic and reconstructive interventions.

The surgical procedures considered included:

1. Mastectomy with immediate reconstruction using an implant or expander: the complete removal of breast tissue, followed by the placement of an implant or expander. This procedure is associated with specific risks, such as seroma formation, infections, or necrosis of the skin flaps, which can affect the healing process.
2. Segmentectomy/quadrantectomy: breast-conserving surgeries.
3. Oncoplastic procedures following local excisions or quadrantectomies: These surgeries combine oncologic resection with plastic surgery techniques to achieve both cancer removal and aesthetic restoration. Oncoplastic procedures may involve reshaping the breast, mobilization of local flaps, or, in cases where the nipple–areola complex cannot be preserved in situ, nipple grafting. Nipple grafting involves harvesting and reattaching the nipple to a new position on the reconstructed breast, which enhances cosmetic outcomes but requires careful management to ensure graft viability. These procedures inherently involve complex tissue manipulation, which can lead to complications such as hematoma, seroma, or asymmetry.
4. Reduction mammoplasty/mastopexy performed concurrently with therapeutic procedures: performed to reshape or reduce the breast while removing cancerous tissue. These procedures involve extensive manipulation, which can lead to risks such as delayed wound healing, nipple necrosis, or wound dehiscence, especially in patients with predisposing factors like comorbidities or a history of smoking.

The protocol involved the prophylactic use of NPWT dressing in high-risk patients instead of standard dressings, with the dressing applied directly in the operating room immediately after skin closure.

Drains placed at the end of the surgery were excluded from the negative pressure dressing and managed independently.

The dressings were checked and replaced within the first two days after surgery to assess wound healing status and the take of any nipple grafts before discharging. Patients were discharged with the NPWT dressing in place and were subsequently seen seven days post-surgery for wound inspection, dressing removal, or a decision to continue therapy for an additional week based on clinical evaluation.

The study assessed the effectiveness of the NPWT dressing system based on various clinical and personalized parameters:

• Postoperative complications: hematomas, necrosis, skin ischemia, wound dehiscence, infection, implant loss, and need for reoperation.
• Healing time: Time to complete wound healing was tracked, alongside molecular markers for wound repair, such as PCR (C-reactive protein), marker of systemic inflammation, which is useful for monitoring the early inflammatory phase of wound healing and glucose levels to evaluate glycemic control, as hyperglycemia is associated with impaired wound healing.
• Cosmetic outcomes: These were evaluated using standardized clinical photography and patient-reported outcomes (PROs) focusing on aesthetic satisfaction.

### Statistical Analysis

Given the small sample size, the study focused on descriptive statistics and trend identification. No *p*-values were calculated, as this was an exploratory study. The analyses focused on data descriptions and the identification of preliminary trends, which require further validation in larger studies.

## 3. Results

### 3.1. Patient Demographics and Risk Factors

**Figure 1:**
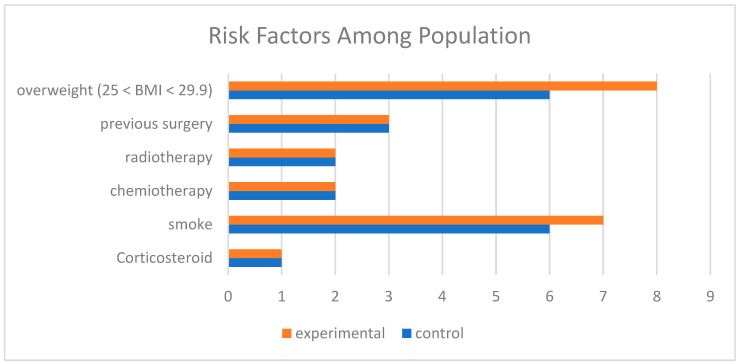
Comparison of risk factors among populations. Legend: Overweight (25 < BMI < 29.9): Indicates the number of participants classified as overweight (BMI between 25 and 29.9) in the experimental group (orange) and control group (blue). Previous Surgery: Participants who underwent prior surgical procedures. Radiotherapy: Participants who received radiotherapy. Chemotherapy: Participants who received chemotherapy. Smoke: Participants with a smoking habit. Corticosteroid: Participants who used corticosteroids. The colors represent the groups: Orange: Experimental group. Blue: Control group.

The study included 22 patients divided into equal groups. The mean BMI was 24.0 (SD = 6.2), ranging from 17.1 to 41.4. All patients presented with at least one risk factor: 50% had a single risk factor, while the remainder had two or more. The most common risk factors were neoadjuvant chemotherapy and smoking, evenly distributed between the two groups. Patient characteristics, including age, BMI, and comorbidities, are summarized in Figure 1.

### 3.2. Surgical Procedures

The majority of patients (50%) underwent implant-based reconstruction. Oncoplastic procedures were performed in 31.8%, while reduction mammoplasty/mastopexy accounted for 13.64%. Quadrantectomies were performed in 4.55% of cases. Surgical complexity was comparable across groups.

### 3.3. NPWT Application and Management

**Figure 2:**
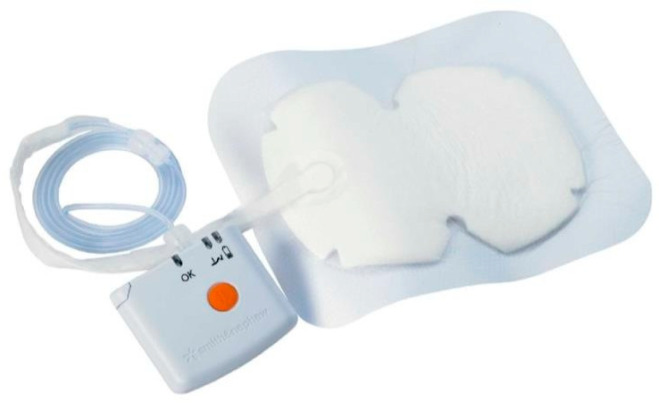
NPWT dressing system.

**Figure 3:**
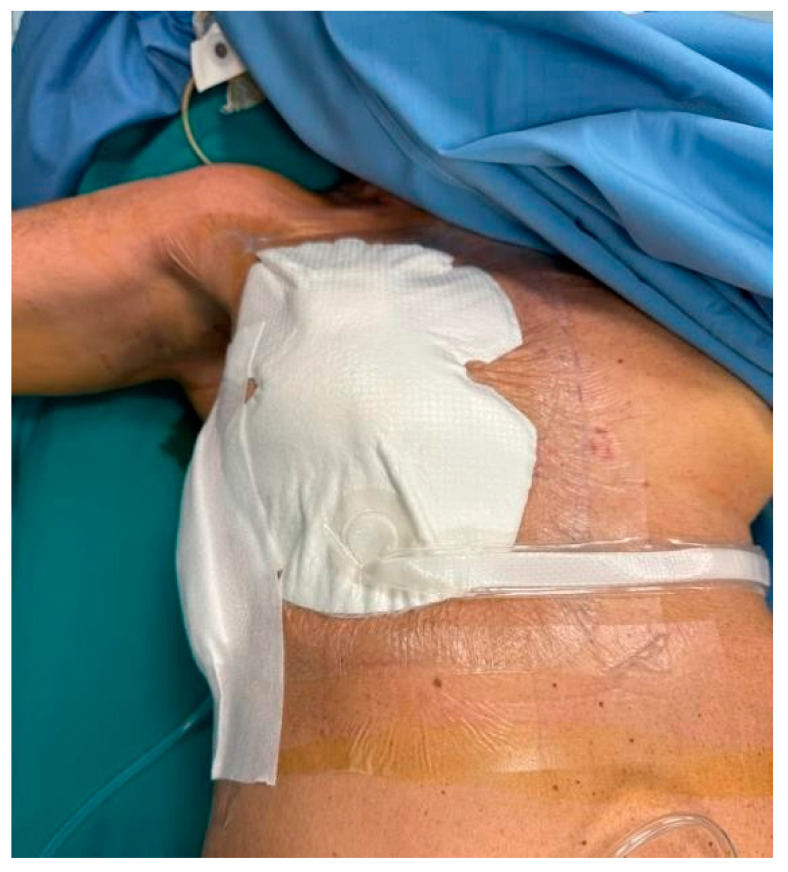
NPWT dressing placement.

NPWT dressings were applied to the experimental group, including bilateral procedures where each breast was assessed separately (Figure 2 and Figure 3). The average duration of NPWT dressing use was 7.15 days (SD = 1.40). Dressings were monitored for adherence, wound healing progression, and any need for reapplication due to complications.

### 3.4. Hospital Stay and Drain Management

The average hospital stay was 3.77 days (SD = 1.79), and drain retention averaged 10.0 days (SD = 6.02). Drain removal criteria were based on output reduction to below 30 cc per 24 h.

### 3.5. Cosmetic and Functional Outcomes

Patient-reported outcomes indicated higher aesthetic satisfaction in the NPWT group, attributed to better wound healing and reduced complications. Standardized clinical photography corroborated these findings, showing improved scar quality and reduced wound tension.

### 3.6. Summary of Findings

The study demonstrated a lower complication rate in the NPWT group (15.4%) compared to the control group (36.4%). Although statistical significance was not assessed due to the sample size, preliminary trends suggest that NPWT dressings may enhance wound healing and reduce complications in high-risk patients. Future studies with larger cohorts are needed to validate these findings.

## 4. Complications

Complications occurred in both groups. In the study group treated with the NPWT dressing, two dressings were applied, and individual breasts were assessed separately. Although the study involved 11 patients in the control group, the total number of breasts assessed was 13, as some patients underwent bilateral procedures. In these cases, each breast was evaluated separately to monitor and report complications for both sides. Among the 13 breasts studied, hematomas were observed in three cases. Notably, both breasts of one patient with bilateral dressing placement experienced significant skin compromise, resulting in a complication rate of 15.4% in this group. The patient’s dressing was reapplied for another seven days, extending the total NPWT duration to 21 days. Complications in both groups were evaluated based on clinical observation and patient reports. In the study group, treated with the NPWT dressing, complications were assessed by regularly monitoring the wounds for signs of infection, hematomas, wound dehiscence, skin ischemia, or other abnormalities. Individual breasts were assessed separately. For example, the patient with bilateral dressing placement was monitored closely for skin integrity, and the dressing was reapplied when significant skin compromise occurred, extending the negative pressure wound therapy (NPWT) duration to 21 days (Table 1).

In the control group, complications were reported in four out of eleven patients, resulting in a complication rate of 36.4%. Two patients experienced local infections requiring oral antibiotics initially but later needed parenteral antibiotics and hospitalization. Both eventually underwent implant removal as part of a salvage-prosthesis protocol. Another patient developed mild wound dehiscence, which was treated locally, but also experienced drug-induced hepatitis after receiving antibiotics for fever. The final significant complication involved surgical wound dehiscence, managed conservatively with topical ointments.

**Table 1:** Correlation between complications and risk factors in the study groups.

	NPWT Dressing YES			NPWT Dressing NO		
	Complications YES	Complications No		Complications YES	Complications NO	
	*n* (%)	*n* (%)	*p*-Value	*n* (%)	*n* (%)	*p*-Value
**Neoadjuvant chemotherapy**			0.128			0.015
Yes	0 (-)	8 (100.0)		0 (-)	6 (100.0)	
No	2 (40.0)	3 (60.0)		4 (80.0)	1 (20.0)	
**Obesity (BMI > 30)**			1.000			-
Yes	0 (-)	3 (100.0)		0 (-)	0 (-)	
No	2 (20.0)	8 (80.0)		4 (36.4)	7 (63.6)	
**Overweight (BMI > 25)**			1.000			1.000
Yes	0 (-)	2 (100.0)		0 (-)	1 (100.0)	
No	2 (18.2)	9 (81.8)		4 (40.0)	6 (60.0)	
**Previous surgery**			1.000			1.000
Yes	0 (-)	2 (100.0)		0 (-)	1 (100.0)	
No	2 (18.2)	9 (81.8)		4 (40.0)	6 (60.0)	
**Previous radiotherapy**			1.000			1.000
Yes	0 (-)	1 (100.0)		0 (-)	1 (100.0)	
No	2 (16.7)	10 (83.3)		4 (40.0)	6 (60.0)	
**Diabetes**			-			0.364
Yes	0 (-)	0 (-)		1 (100.0)	0 (-)	
No	2 (15.4)	11 (84.6)		3 (30.0)	7 (70.0)	
**Smoke**			0.462			0.545
Yes	2 (28.6)	5 (71.4)		3 (50.0)	3 (50.0)	
No	0 (-)	6 (100.0)		1 (20.0)	4 (80.0)	
**Corticosteroid**			1.000			0.364
Yes	0 (-)	1 (100.0)		1 (100.0)	0 (-)	
No	2 (16.7)	10 (83.3)		3 (30.0)	7 (70.0)	
**Number of risk factors**			0.462			0.727
1	2 (33.3)	4 (66.7)		3 (50.0)	3 (50.0)	
2	0 (-)	3 (100.0)		1 (25.0)	3 (75.0)	
3	0 (-)	4 (100.0)		0 (-)	1 (100.0)	

## 5. Discussion

The findings of this preliminary study highlight the potential benefits of prophylactic Negative Pressure Wound Therapy (NPWT) using the NPWT dressing in reducing postoperative complications in high-risk patients undergoing oncoplastic and reconstructive breast surgery. Specifically, the complication rate observed in the NPWT dressing group (15.4%) was significantly lower than that in the Standard of Care (SOC) group (36.4%). Major complications, such as infections requiring hospitalization and implant loss, occurred exclusively in the SOC group. These results align with previous studies that suggest NPWT can reduce the risk of surgical site infections (SSIs) and wound dehiscence by promoting optimal wound healing and maintaining a sterile environment [16,17].

The effectiveness of the NPWT dressing likely stems from its ability to reduce wound exudate, enhance tissue perfusion, and prevent seroma formation. These mechanisms have been supported by studies like those of Sogorski et al. (2018), which demonstrate NPWT’s ability to improve microcirculation and reduce tissue edema, both of which are essential for promoting healing in complex wounds [18]. This physiological advantage is particularly relevant for high-risk populations, such as those with obesity, smoking history, and prior radiotherapy, which significantly impair natural wound healing [19].

Our results are consistent with findings from other studies in breast surgery. Karolina Pieszko et al. (2023) reported reduced wound complications following breast reconstruction when NPWT was applied [18]. Similarly, Tommaso Fogacci et al. (2020) demonstrated that NPWT dressing, when used as a prophylactic NPWT in high-risk patients, helps to manage and decrease the incidence of wound infection and ischemia. Based on these findings, NPWT dressing seems to be appropriate even for breast surgery in all patients whose overall condition warrants the use of such a device. [20,21]. These findings reinforce the growing body of evidence supporting NPWT as a valuable adjunct to postoperative care in breast surgery.

One notable challenge associated with NPWT, including the use of the NPWT dressing, is its higher initial cost compared to traditional SOC dressings. Nevertheless, this expense may be offset by reduced hospital stays, fewer readmissions, and avoidance of costly reoperations due to wound complications.

The small sample size represents a significant limitation of this study, restricting the generalizability of the findings. Additionally, the retrospective design further constrains the strength of the conclusions. Future research should explore the long-term benefits of NPWT and its potential integration with other personalized medicine approaches, such as pharmacogenomics, to refine postoperative care strategies.

Further research is also required to identify the optimal duration of NPWT application and determine which surgical procedures benefit most from this intervention. The role of NPWT in multimodal wound care strategies—such as combining NPWT with advanced wound monitoring technologies or adjunctive therapies—should also be explored. Integrating these technologies into clinical protocols may enhance patient outcomes, further reducing complications and promoting faster recovery.

Furthermore, the integration of genetic testing and biomarker profiling could further enhance the personalization of NPWT. Identifying genetic markers related to wound healing, inflammation, and tissue regeneration could help tailor NPWT protocols to individual patients, potentially improving outcomes in a more targeted and cost-effective manner.

## 6. Conclusions

This preliminary study highlights the potential of NPWT dressings to improve postoperative outcomes in high-risk patients undergoing oncoplastic and reconstructive breast surgery. While the results are promising, the small sample size and the preliminary nature of the research limit the generalizability of the conclusions. Future studies with larger sample sizes are essential to confirm the effectiveness of NPWT in more diverse clinical settings.

In addition to confirming the efficacy of NPWT, future research should focus on understanding the molecular mechanisms underlying its effectiveness, exploring long-term outcomes, and conducting cost-effectiveness analyses to evaluate its broader applicability in clinical practice. Furthermore, integrating NPWT with personalized medicine approaches, such as pharmacogenomics and genetic considerations, could offer significant potential for refining treatment protocols and enhancing patient recovery.

## Data Availability

The data presented in this study are available in this article.

## Abbreviations

The following abbreviations are used in this manuscript:

NPWT	Negative Pressure Wounds Therapy
SOC	Standard of Care
